# Variation of *Mycobacterium tuberculosis* Antigen-Specific IFN-γ and IL-17 Responses in Healthy Tuberculin Skin Test (TST)-Positive Human Subjects

**DOI:** 10.1371/journal.pone.0042716

**Published:** 2012-08-06

**Authors:** Lin Fan, He-ping Xiao, Zhong-yi Hu, Joel D. Ernst

**Affiliations:** 1 Division of Infectious Diseases, New York University School of Medicine, New York, New York, United States of America; 2 Tuberculosis Center for Diagnosis and Treatment, Shanghai Pulmonary Hospital, Tongji University School of Medicine, Shanghai, China; National Institute for Infectious Diseases (L. Spallanzani), Italy

## Abstract

**Objective:**

To determine the variation of IFN-γ and IL-17 responses to *M. tuberculosis* antigens in healthy TST+ humans.

**Methods:**

We isolated peripheral blood mononuclear cells from 21 TST+ healthy adults, stimulated them with phytohemagglutinin (PHA), PPD, Ag85B, ESAT-6, and live *M. bovis* BCG, and assayed IFN-γ and IL-17 secretion by ELISA in supernatants after 24 or 72 hours of incubation respectively.

**Results:**

As in other studies, we found a wide range of IFN-γ responses to *M. tuberculosis* antigens; the variation significantly exceeded that observed in the same donors to the polyclonal T cell stimulus, phytohemagglutinin (PHA). In addition, we assayed IL-17 secretion in response to the same stimuli, and found less subject-to-subject variation. Analysis of the ratio of IFN-γ to IL-17 secretion on a subject-to-subject basis also revealed a wide range, with the majority of results distributed in a narrow range, and a minority with extreme results all of which were greater than that in the majority of subjects. The data suggest that study of exceptional responses to *M. tuberculosis* antigens may reveal immunologic correlates with specific outcomes of *M. tuberculosis* infection.

**Conclusion:**

Variation of IFNγ and IFN-γ/IL-17 responses to mycobacterial antigens exceeds that of responses to the polyclonal stimulus, PHA, in TST positive healthy humans. This indicates a quantitative spectrum of human immune responses to infection with *M. tuberculosis*. Since the outcome of human infection with *M. tuberculosis* varies greatly, systematic study of multiple immune responses to multiple antigens is likely to reveal correlations between selected immune responses and the outcomes of infection.

## Introduction

Tuberculosis is one of the leading infectious diseases in the world; the estimated total number of incident cases of tuberculosis worldwide rose to 9.4 million in 2009—more than at any other time in history, resulting in an estimated 1.7 million deaths each year [1]. *Mycobacterium tuberculosis* can establish lifelong persistent infection in immunocompetent hosts, reflecting a remarkable adaptation to its human hosts. Despite the inability of adaptive immune responses to eradicate *M. tuberculosis*, CD4 T cells play a major role in controlling *M. tuberculosis* infection in humans, as indicated by the observations that HIV-infected, CD4 T cell-deficient individuals are more susceptible to tuberculosis than are HIV-uninfected individuals. Moreover, the incidence of active tuberculosis in HIV-infected individuals is inversely proportion to the number of CD4 T cells in peripheral blood [2], [3].

In addition to their roles in providing protective immunity against tuberculosis, *M. tuberculosis* antigen-specific CD4 T cells may also contribute to immunopathology in the lungs and other organs [4], [5]. For example, multiple studies have revealed that, in HIV-infected patients with active tuberculosis, the frequency of cavitary lung lesions is directly proportional to the number of the number of peripheral blood CD4 T cells at the time of TB diagnosis [2], [6]. Furthermore, one study has revealed that distinct functional subsets of CD4 effector T cells may predominate at the site of infection in patients with cavitary and non-cavitary pulmonary TB: Th1 cells were more common in bronchial lavage of patients with non-cavitary TB, while Th2 cells were more abundant in patients with cavitary TB [7]. In contrast, another study found that lavage fluid of cavitary TB lesions contained a predominance of neutrophils [8], which is consistent with several potential mechanisms, including a dominant Th17 response [9]. Finally, recent studies in mice infected with *M. tuberculosis* have revealed that dysregulation of CD4 T cell responses causes severe lung inflammation and contributes to mortality [9], [10]. In particular, excessive IL-17 secretion has been found to be associated with severe lung inflammation [9].

During primary immune responses, naïve CD4 T cells can differentiate into Th1, Th2, or Th17 effector cells, or into regulatory (Treg) cells; this is driven by distinct cytokines present during proliferation and differentiation of antigen-activated T cells [11], [12]. These subsets of T helper cells have distinct functions. In particular, IFN-γ is the signature cytokine of Th1 cells, and can activate mycobactericidal mechanisms in macrophages and restrict progressive growth and dissemination of *M. tuberculosis* infection. IFN-γ also has potent immunoregulatory effects that attenuated other T cell responses [9] and that regulate neutrophil-mediated inflammation [13]. IL-17, produced by Th17 cells, is a potent pro-inflammatory cytokine, as it induces expression of G-CSF, resulting in increased production of neutrophils and induces expression of selected neutrophil chemokines. Although IL-17 has been shown to have beneficial effects in mice during early infection with *M. tuberculosis* [14], [15], excessive IL-17 responses are detrimental to mice infected with *M. tuberculosis* [9], [16]. In addition to being produced by Th17 cells, IL-17 can be produced by other cells, including γδ T cells [17], which may play a role in control of *M. tuberculosis* infection through the induction and formation of mature granulomas [13]. In humans infected with M. tuberculosis, IL-17 is expressed by antigen-specific CD4+ T cells, a subset of these may also express other cytokines, including IFN-γ, TNF, and/or IL-2 [18]. Th1 and Th17 responses can cross-regulate each other during *M. tuberculosis* infection; this may be important for limiting immunopathologic consequences of infection [9], [19], [20].

In light of evidence that CD4 T cells may contribute to both control of *M. tuberculosis* as well as to the immunopathology that contributes to morbidity and mortality in TB, there is considerable interest in identifying differential CD4 T cell responses that are associated with beneficial and detrimental outcomes. The present study was designed to test the overall hypothesis that humans infected with *M. tuberculosis* or vaccinated with *M. bovis* BCG generate a range of Th1 and Th17 responses to *M. tuberculosis* antigens. As an initial test of this hypothesis, we quantitated interferon gamma (IFN-γ) and IL-17 secretion after stimulation with selected *M. tuberculosis* antigens by PBMC from tuberculin skin test-reactive (TST+) healthy individuals. We were especially interested in determining the intersubject variation in the relationship between the quantity of IFN-γ and IL-17 released in response to *M. tuberculosis* antigens.

## Results

### Characteristics of subjects

Twenty one tuberculin skin test positive (≥5 mm induration; TST+) healthy adults in New York City gave informed consent and donated venous blood for this study. The subjects ranged in age from 21 to 62 years old (mean 34.1 years), and were diverse in their countries of birth, which included Turkey, Peru, Pakistan, South Korea, Vietnam, Nigeria, Russia, China, Brazil, Albania, Uraguay, Jamaica, the Philippines, and the USA. Four of the subjects (donors 14, 17, 20, and 40) had been treated previously for latent TB infection; seven (donors 16, 24, 26, 31, 32, 36, and 40) reported having been vaccinated with BCG. Three (donors 15, 36, and 40) had >5 but <10 mm induration in response to intradermal PPD. None of the subjects had a history of active tuberculosis, and none had evidence of HIV infection.

### Variation in IFN-γ responses

**Figure 1:**
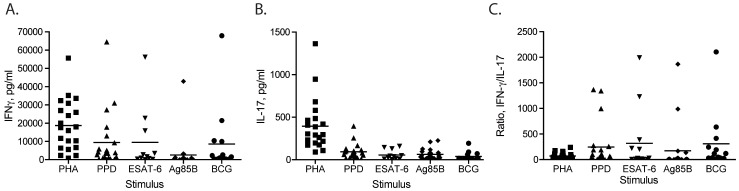
Variation of IFN-γ and IL-17 responses to mycobacterial antigens. PBMC were incubated with the indicated stimuli for 24 h (for IFN-γ responses) or 72 h (for IL-17 responses); cytokine secretion was assayed by ELISA. (A) IFN-γ secretion in response to PHA, PPD, ESAT-6, Ag85B, or live *M. bovis* BCG. Each symbol reflects responses of a single subject whose responses were assayed in triplicate. The horizontal bar indicates the mean value for the group. (B) IL-17 secretion in response to PHA, PPD, ESAT-6, Ag85B, or live *M. bovis* BCG. Each symbol reflects responses of a single subject whose responses were assayed in triplicate. The horizontal bar indicates the mean value for the group. (C) Ratio of IFN-γ to IL-17 secretion in response to the stimuli in panels A and B. Ratios were calculated using the mean value for IFN-γ and for IL-17 for each subject; the horizontal bar indicates the mean value for the group of subjects.

Since IFN-γ has been widely studied, and since it is essential for control of tuberculosis in mice and in humans, we examined its secretion in response to selected stimuli. As shown in Figure 1A and Table 1, there was a large intersubject range in the amounts of IFN-γ secreted in response to the mycobacterial antigens Purified Protein Derivative (PPD), ESAT-6, and Ag85B, as well as with whole, live, *Mycobacterium bovis* BCG. For example, PPD stimulated IFN-γ secretion in all 21 subjects, but the amounts varied between individuals over a 500-fold range, from 128 to 64,569 pg/ml (Figure 1A and Table 1). Likewise, IFN-γ responses to Ag85B were detectable in 19 of 21 subjects and varied over a 1,525-fold range, from 28.1 to 42,879 pg/ml. ESAT-6 stimulated detectable quantities of IFN-γ in 14 of 21 subjects, consistent with our inclusion of subjects with a history of BCG vaccination. In the 14 subjects that responded to ESAT-6, IFN-γ secretion varied over an even wider, 4,352-fold range, from 12.9 to 56,147 pg/ml. It is notable that among the donors, the largest quantities of IFN-γ secreted in response to PPD, Ag85B, ESAT-6, and live BCG all occurred in the same subject (donor 12, Table 1), although the lowest IFN-γ responses to these stimuli occurred in 4 different subjects (donors 11, 26, 16, and 23, respectively; Table 1). Notably, despite the heterogeneity of the subjects with respect to their BCG vaccination status or treatment for latent TB infection, the responses of individuals in these specific groups did not account for either the highest or the lowest responses to any of the stimuli, with the exception that cells from 4 of the 7 subjects with a history of BCG vaccination did not respond to stimulation with ESAT-6.

**Table 1:** IFN-γ and IL-17 responses in healthy TST-positive donors.

Subject	Unstimulated control			PHA			PPD			ESAT-6			Ag85B			BCG		
	IFNγ	IL17	Ratio	IFNγ	IL17	Ratio	IFNγ	IL17	Ratio	IFNγ	IL17	Ratio	IFNγ	IL17	Ratio	IFNγ	IL17	Ratio
2	320	15.3	21.2	26930	217.1	125.5	959.5	129.3	8.6	1602.6	8.3	219.5	3109	117	27.9	9942.7	5.9	2105.3
7	0	3.3	0	25951.4	262.4	98.7	5873.3	93.7	65.9	3426.0	157.7	23. 8	*0*	*76.5*	*0*	2661.5	14.7	193.2
11	*2.04*	*0*	*NV*	782.0	66.6	13.1	128.2	36.2	3.7	*0*	*0*	*NV*	*1.6*	*15.4*	*0.21*	*378.2*	*0.99*	*0*
12	16.4	1.2	14.2	30910	174.6	176.4	64569	54.9	1344.9	56147	35.3	1990.5	42879	44.7	989.2	67865	193.3	415.6
14	0	0.09	31.2	18020	387.4	46.9	1502.2	395.3	3.8	*0.68*	*69.2*	*0.03*	381.4	209.9	1.9			
15	0	0.5	0	6686.9	484.1	13.8	3478.4	13.5	256.6	854.7	144.0	5.9	797.2	225.9	3.8			
16	0	2.8	0	2255.3	171.8	13.3	1303.9	17.3	97.9	*0*	*2.4*	*0*	28.1	11.2	2.8			
17	0	2.8	0	6343.4	90.3	71.9	1054.9	22.1	48.9	*0*	*2.8*	*0*	145.4	15.5	9.8			
19	0	5.0	0	55626	232.6	240.9	2744.8	101.9	27.8	*0*	*12.9*	*0*	1190	96.5	13.0			
20	0	6.2	0	10338	464.9	22.9	4205.7	114.6	35.3	333.7	42.6	8.4	47.7	32.7	2.1			
22	0	1.4	0	11672	291.2	40.4	437.9	8.5	53.6	*2.1*	*5.6*	*0.53*	72.4	22.6	3.311			
23	0	2.2	0	17512	108.5	161.2	9322.4	9.3	997.4	2700.4	7.3	387.4	787.1	6.4	136.2	130	4.9	23.5
24	0	1.7	0	10300	196.9	53.8	400.8	104.4	3.1	*0*	*16.1*	*0*	531.9	126.5	6.6	1566.3	72.6	30.4
26	0	3.56	0	24183	581.1	41.7	13017	72.6	189.8	12.9	12.8	1.2	853.8	81.8	11.2	361.8	19.1	16.6
27	0	2.75	0	32359	295.2	110.8	17800	67.0	277.4	594.7	48.4	13.5	171.3	36.2	5.6	2224.6	61.6	34.9
31	98.1	1.3	83.1	15955	681.7	32.166	3816.0	84.3	40.1	57.7	2.3	27.2	199.3	46.8	4.9	964.7	11.4	119.8
32	15.8	13.9	1.2	35153	1363.4	32.3	5103	45.2	109.4	17.7	6.4	3.6	373.8	14.7	26.4	458.5	18.2	37.2
33	290.8	18.6	83.2	33582	419.1	81.6	27342.7	20.1	1367.5	15772.3	12.9	1227.9	3099	18.6	167.8	10383	16.6	635.8
36	0	4.7	0	5623.3	303.8	18.6	241.8	61.6	3.9	*0*	*6.3*	*0*	40.8	77.8	0.7	361.3	10.2	48.4
38	0	1.5	0	19954	406.9	49.7	30998	167.3	190.3	22702	129.1	200.9	44.4	38.0	1.1	21422	92.3	244.80
40	15.6	4.2	3.2	2311	946.7	2.4	3530	80.4	43.1	203.8	24.9	8.3	85.8	19.9	4.6	676.6	6.1	118.5

IFNγ and IL-17 values are expressed in pg/ml. Ratio indicates IFN-γ/IL-17. NV = No value; not calculated due response of IFN-γ or IL-17 below the limit of detection.

To determine whether the wide range of intersubject variation in IFN-γ secretion simply reflected variation of the number of viable T cells in the individual PBMC samples and/or a general property of the T cells in the individual subjects, we examined IFN-γ secretion in response to the polyclonal T cell stimulus, phytohemagglutinin (PHA). This also revealed a range of IFN-γ responses. However, the variation was less than for any of the mycobacterial antigens, varying only 70-fold (from 782 to 55,626 pg/ml) in the 21 subjects. Moreover, the subjects whose cells secreted the largest quantities of IFN-γ in response to PHA were not the same as those with the largest responses to mycobacterial antigens. These results indicate a very large variation in intersubject responses to *M. tuberculosis* antigens, and that the variation is much wider than to a polyclonal T cell stimulus.

### Variation in IL-17 responses

To further determine the extent to which T lymphocytes in individual TST+ subjects vary in their responses to mycobacterial antigens, we assayed interleukin-17A (IL-17) secretion in response to the same stimuli as used for IFN-γ responses. This also revealed a range of individual responses, varying 46-, 35-, 68.5-, and 39-fold, to PPD, Ag85B, ESAT-6, and live BCG, respectively (Figure 1B and Table 1). By comparison, individual IL-17 responses to PHA varied over a narrower, 20.5-fold range. It is also noteworthy that the individuals whose IFN-γ responses represented the high and low extremes in response to the selected mycobacterial antigens were not the same as those with the extremes of IL-17 responses (Table 1). When responses to PPD were analyzed for the 21 donors, there was no correlation between IFN-γ and IL-17 responses (Spearman r value, 0.055; p = 0.81).

### Comparison of IFN-γ and IL-17 responses within and between donors

The separate analyses of IFN-γ and IL-17 responses indicated considerable variation between individuals for secretion of these two cytokines in response to *M. tuberculosis* antigens, and suggested that the two responses were not fully concordant. Therefore, we examined the range of variation in the ratio of the amounts of IFN-γ and IL-17. This also revealed considerable intersubject variation, with ratios of IFN-γ/IL-17 secretion varying 441- , 1,412- , 1,658- and 127-fold for PPD, Ag85B, ESAT-6, and BCG, respectively (Figure 1C and Table 1). Consistent with the smaller variation in IFN-γ and IL-17 responses to PHA compared to responses to mycobacterial antigens, the ratios of IFN-γ/IL-17 varied over a 100-fold range (Figure 1C and Table 1).

Examination of the ratios of IFN-γ/IL-17 in response to individual antigens revealed that 3 subjects (donors 12, 23, and 33; Table 1) exhibited the highest ratios to all 3 of the soluble *M. tuberculosis* antigens (PPD, Ag85B, and ESAT-6). Two of these (subjects 12 and 23) also exhibited the highest ratios in response to PHA, while the ratio of subject 33 ranked seventh among the subjects in response to PHA. The heterogeneity in donors with respect to BCG vaccination status or history of treatment for latent TB infection did not account for these extremes, as the responses in these individuals were in the intermediate range for the entire group of donors.

The analysis of intersubject variation of IFN-γ , IL-17, and the ratio of IFN-γ/IL-17 suggested that, while the results for most of the subjects clustered in a narrow range, the results for several of the subjects were outside of this range, and exceeded it by a considerable amount (Table 2). To determine whether the three subjects with the highest ratios of IFN-γ/IL-17 represent true extremes, we examined them using previously-described criteria [21]. This revealed that when responses to PPD or Ag85B were considered, all three subjects met the criteria for extreme responses (Table 3). The ratios of two of the subjects met the criteria for extreme responses to ESAT-6, and one of the three met the criteria when BCG was used as the stimulus.

**Table 2:** Descriptive Statistics of IFN-γ/IL-17 ratios.

Stimulus:	PHA	PPD	ESAT-6	Ag85B	BCG
Number of subjects studied per stimulus:	21	21	13	19	13
Minimum	2.400	3.100	1.200	0.7000	16.60
25%ile	21.00	18.30	7.150	3.300	32.50
Median	47.00	54.00	24.00	6.600	118.0
75%ile	105.0	223.5	303.5	28.00	330.5
Maximum	241.0	1367	1990	1865	2105

**Table 3:** Analysis of Extreme IFN-γ/IL-17 Responses.

	PPD (cutoff = 307.8)	Ag85B (cutoff = 37.1)	ESAT-6 (cutoff = 444.5)	BCG (cutoff = 446.3)
Subject				
12	1344	989	1990	416
23	997	136	387	24
33	1367	168	1228	6358

The cutoff used to define extreme values was calculated using the equation Q3+1.5 (Q3-Q1), where Q3 and Q1 are the third quartile (75%ile) and first quartile (25%ile) of the results from all of the responding subjects. Ratios above this calculated value for each stimulus were defined as extreme.

### Stimulus-by-stimulus comparison of IFN-γ/IL-17 responses

In addition to the variation in IFN-γ and IL-17 responses by the cells of individual subjects, we also noted a range of IFN-γ and IL-17 ratios in response to individual mycobacterial stimuli. For the crude antigen mixtures represented by PPD and live *M. bovis* BCG, the median IFN-γ/IL-17 ratios varied over approximately a 2-fold range (54–118; Table 2), while for the two purified protein antigens, Ag85B and ESAT-6, the median ratios varied nearly 4-fold (6.6–24.0; Table 2). These results suggest that individual *M. tuberculosis* antigens may induce qualitatively distinct T cell responses, perhaps due in part to the differences in expression patterns of the genes encoding these antigens [22].

## Discussion

The aim of this study was to quantitate the variation of IFN-γ/IL-17 in response to *M. tuberculosis*-specific antigens in healthy TST+ adults. We found extensive variation in the quantities of IFN-γ and IL-17 secreted in response to PPD, Ag85B, ESAT-6, and live *M. bovis* BCG. We also found that the variation in IFN-γ and IL-17 responses to mycobacterial antigens was greater than that to the polyclonal T cell stimulus, PHA, indicating that the variation in intersubject responses was not a general property of T cell responses of the individual subjects.

While this study revealed wide variation in IFN-γ and IL-17 responses to PPD, Ag85B, ESAT-6, and live BCG in a group of subjects, it also revealed that a subset of subjects exhibits extreme responses, reflected by secretion of large quantities of IFN-γ, and high ratios of IFN-γ/IL-17 secretion. This is particularly remarkable, since all of the subjects were healthy at the time of the analysis, supporting the model that latent TB infection represents a spectrum of host and bacterial interactions [23]. Additional longitudinal analyses that consider multiple T cell responses to multiple mycobacterial antigens and correlate them with the outcomes of infection (that is, prolonged latent TB infection, or progression to active TB disease) may provide insight into the adaptive immune responses that provide durable control of *M. tuberculosis*, especially if they take into account the effects of variations in the infecting bacteria and in the genetic backgrounds of the human subjects involved [24]. Furthermore, the temporal characteristics of bacterial antigen gene expression during the course of infection may contribute to variations in the T cell responses to individual antigens [22], [25], [26]. For example, antigens expressed at different times after infection may occupy dendritic cells in distinct inflammatory environments that cause them to drive differentiation of the responding antigen-specific T cells toward Th1 or Th17 pathways.

Since human CD4 T cells are implicated in immunopathology as well as in protective immunity in tuberculosis, identification of correlates of CD4 T cell-mediated immunity, and the design of improved TB vaccines, is proving to be a challenge. In particular, the number of peripheral blood CD4 T cells at the time of TB diagnosis in HIV-TB coinfected individuals correlates closely with the likelihood that a patient will have cavitary tuberculosis [2]. Although the pathogenesis of the lung tissue damage that underlies cavitary TB is poorly understood, it appears that CD4 T cells contribute, directly or indirectly. Since cavitary TB causes significantly more secondary cases than does non-cavitary TB (reviewed in [2]), characterization of the antigen specificity and effector functions of CD4 T cells in persons that progress from latent TB to cavitary or noncavitary TB can have considerable public health importance, as identification and treatment of those at highest risk may provide a cost-effective approach to decreasing TB transmission. Our finding that healthy TST+ individuals exhibit wide variation in the amounts of IFN-γ and IL-17 secreted in response to mycobacterial antigens suggests that analysis of multiple T cell effector functions in response to mycobacterial antigens may reveal correlates of specific outcomes of *M. tuberculosis* infection. Such studies should also take into account the potential impact of infection with distinct strains of *M. tuberculosis*, especially in individuals of distinct genetic backgrounds [24]. The results reported here also suggest that analysis of subjects with extreme responses might be especially valuable in revealing correlates of protective immunity and immunopathology.

The present study has several limitations. First, as a pilot study in healthy individuals, it does not provide the opportunity to compare specific T cell responses with clinical or pathological outcomes. Nevertheless, it provides data on the spectrum and extent of variation of IFN-γ and IL-17 responses to serve as the basis for further studies of subjects with other clinical phenotypes. Further studies are needed to extend the present findings, to determine whether the wide donor-to-donor variation in cytokine secretion observed in this study represent differences in the number of responding cells, the amount of cytokine synthesized per cell, or both. Second, even though all of our subjects were healthy adults, they included subjects that had received BCG vaccine, and they consisted of ethnically-diverse individuals that are likely to be latently infected with *M. tuberculosis* strains from distinct genetic lineages [27]. Moreover, they may also vary in the duration between their initial infection with *M. tuberculosis* and/or BCG vaccination and the time of their blood donation. Although the natural history of antigen-specific T cell responses to mycobacterial antigens has not been characterized in detail outside the context of vaccine trials, this is a variable that may have contributed to our findings.

In conclusion, this study provides evidence for a wide range of IFN-γ and IL-17 responses to *M.tuberculosis* specific antigens in TST+ adults, and indicates that in a population certain individuals exhibit extreme T cell responses to mycobacterial antigens. We anticipate that these findings will be valuable in characterizing T cell responses to *M. tuberculosis* antigens in other contexts, and will provide the basis for expanded studies to identify correlates of protective and pathologic immune responses in human tuberculosis.

## Methods

### Human subjects

During a 4 month period, healthy healthcare and research workers at the New York University Medical Center were screened by tuberculin skin testing and offered enrollment in this study if they responded with ≥5 mm induration 48–72 hours after intradermal injection of 5 TU of PPD. None of the subjects were taking a regularly prescribed medication such as steroids or immunosuppressive agents; individuals with HIV infection were excluded from the present study. All of the the subjects were >18 years of age and provided written informed consent. Information on BCG vaccination status was requested but was not confirmed by independent means; IGRA testing was not routinely available at our medical center during the period of this study. Written informed consent was obtained from all subjects, and all procedures were performed in accord with a protocol approved by the New York University Medical Center Institutional Review Board.

### Isolation and stimulation of PBMC

Venous blood samples (30 ml) were collected in heparin tubes; peripheral blood mononuclear cells (PBMC) were isolated within 3 hours after blood collection. PBMC isolation was performed by density gradient centrifugation using Ficoll-Hypaque (Sigma-Aldrich Histopaque-1077) following the manufacturer's instructions. After washing the cells, 3×10^5^ cells per well were cultured in 200 µL of RPMI-1640 (GIBCO, Invitrogen) with 10% heat inactivated pooled human AB serum, penicillin (50 U/ml), streptomycin (50 µg/ml), 2 mM L-glutamine and 10 mM HEPES using 96-well round-bottom plates (Costar). PBMC were then stimulated with PHA (5 µg/ml), BCG (nominal MOI 1∶1), PPD (purified protein derivative, 10 µg/ml, Statens Serum Institute Demark), native *M. tuberculosis* Ag85B (5 µg/ml, BEI Resource, USA), recombinant ESAT-6 (5 µg/ml, Statens Serum Institue, Demark), or medium alone as a negative control.

### Cytokine analyses in culture supernatants by ELISA

Cells were cultured and stimulated with the aforementioned antigens and controls for 24 hours (for IFN-γ responses) or 72 hours (for IL-17 responses) at 37°C with 5% CO_2_. Supernatants were collected and stored at −70°C until the time of assay. The concentration of IFN-γ was determined using the Human IFN-γ ELISA Set (BD Biosciences, San Diego, CA,USA), after diluting the supernatants sufficiently to yield results within the linear range of the assay (routine dilutions were: PHA-stimulated samples, 1∶200; BCG 1∶20; PPD, 1∶10–1∶50 and Ag85B stimulated samples at 1∶2) Supernatants from negative controls or ESAT-6 stimulated samples were not diluted, except for some samples that contained high concentrations of IFN-γ after ESAT-6 or Ag85B stimulation, which were reassayed after dilution from 1∶10–1∶200. IL-17 concentrations in supernatants were quantitated using the Human IL-17 ELISA Set (eBioscience, USA) in supernatant after 72 hours incubation. Supernatants from PHA-stimulated samples were diluted 1∶2; all other assays were performed on undiluted supernatants. All assays were done in triplicate. Plates were read at 450 nm within 30 minutes of the end of the assay, using an ELISA plate reader.

### Statistical analyses

Descriptive statistics were obtained, and Spearman correlation was determined using Prism 4 for the Macintosh (GraphPad Software, USA). Determination of extreme values was performed as described in [21], and is detailed in the legend for Table 3.
